# iMM1865: A New Reconstruction of Mouse Genome-Scale Metabolic Model

**DOI:** 10.1038/s41598-020-63235-w

**Published:** 2020-04-10

**Authors:** Saeideh Khodaee, Yazdan Asgari, Mehdi Totonchi, Mohammad Hossein Karimi-Jafari

**Affiliations:** 1 Department of Bioinformatics, Institute of Biochemistry and Biophysics, University of Tehran, Tehran, Iran; 2 Department of Medical Biotechnology, School of Advanced Technologies in Medicine, Tehran University of Medical Sciences, Tehran, Iran; 3 Department of Stem Cells and Developmental Biology, Cell Science Research Center, Royan Institute for Stem Cell Biology and Technology, ACECR, Tehran, Iran; 4 Department of Genetics, Reproductive Biomedicine Research Center, Royan Institute for Reproductive Biomedicine, ACECR, Tehran, Iran

**Keywords:** Biochemical reaction networks, Systems biology

## Abstract

Since the first in silico generation of a genome-scale metabolic (GSM) model for *Haemophilus influenzae* in 1999, the GSM models have been reconstructed for various organisms including human and mouse. There are two important strategies for generating a GSM model: in the bottom-up approach, individual genomic and biochemical components are integrated to build a GSM model. Alternatively, the orthology-based strategy uses a previously reconstructed model of a reference organism to infer a GSM model of a target organism. Following the update and development of the metabolic network of reference organism, the model of the target organism can also be updated to eliminate defects. Here, we presented iMM1865 model as an orthology-based reconstruction of a GSM model for *Mus musculus* based on the last flux-consistent version of the human metabolic network, Recon3D. We proposed two versions of the new mouse model, iMM1865 and min-iMM1865, with the same number of gene-associated reactions but different subsets of non-gene-associated reactions. A third extended but flux-inconsistent model (iMM3254) was also created based on the extended version of Recon3D. Compared to the previously published mouse models, both versions of iMM1865 include more comprehensive annotations of metabolites and reactions with no dead-end metabolites and blocked reactions. We evaluated functionality of the models using 431 metabolic objective functions. iMM1865 and min-iMM1865 passed 93% and 87% of the tests, respectively, while iMM1415 and MMR (another available mouse GSM) passed 80% and 84% of the tests, respectively. Three versions of tissue-specific embryo heart models were also reconstructed from each of iMM1865 and min-iMM1865 using mCADRE algorithm with different thresholds on expression-based scores. The ability of corresponding GSM and embryo heart models to predict essential genes was assessed across experimentally derived lethal and viable gene sets. Our analysis revealed that tissue-specific models render much better predictions than GSM models.

## Introduction

A genome-scale metabolic (GSM) model is a comprehensive model for metabolism of an organism that includes all known chemical reactions and their corresponding associated genes^1^. For each enzyme-associated reaction in a GSM model, a gene-protein-reaction (GPR) rule describes the relationship between necessary genes encoding the enzyme that catalyses this reaction^2,3^. These GPR associations enable GSM models to be used for prediction of phenotypic consequences of genetic perturbations^4^. For multicellular organisms, these rules allow integration of gene or protein expression data with a GSM model and reconstruction of cell- and tissue-specific models^4,5^. Also, GPR rules are a fundamental component of a GSM model of a reference organism to reconstruct new GSM models of another target organism by homologous gene mapping^6^.

To reconstruct GSM models, a traditional approach is a bottom-up method that integrates individual genomic and biochemical components to achieve a consistent model^1,3^. The first step is to automatically generate a draft reconstruction based on extracting metabolic function from the genome annotation and biochemical reaction data within related databases such as KEGG^7^, BioCyc^8^, and BRENDA^9^. The second step is to manually curate the draft reconstruction using literature information. In the final step, evaluation process is performed to examine the model for mass and charge balances, existence of dead-end metabolites and blocked reactions, and passing functional metabolic tasks^1^. The two last steps are iterated until the resulting model becomes as gap-free and consistent as possible. Based on this approach, the first comprehensive GSM models of human, Recon1^10^ and Edinburgh Human Metabolic Network (EHMN)^11^, were generated by different groups in 2007. To date, various human models have been presented to improve these primary models. The most recent of these networks are the other versions of Recon including Recon2.02^12^, and Recon3D^13^, and also recent versions of Human Metabolic Reaction (HMR) models; HMR1^14^ and HMR2^15^. In the case of mouse models, Quek and Nielsen^16^ created a compartmentalized mouse model from available online metabolic databases. Their model contains 2037 reactions, 2110 metabolites and 1399 genes. Two years later Selvarasu *et al*.^17^ extended an older model by including additional information on GPR associations and improving the network connectivity in many biosynthetic pathways. The resulting model contains 1494 reactions, 1162 metabolites and 724 genes. Both these models only include three compartments (cytosol, mitochondria and extracellular space) while more modern models have additional compartments including the Golgi apparatus, lysosome, nucleus, endoplasmic reticulum and peroxisome. In a large-scale generation of computational models, the Path2Models project also generated a mouse model that is an automatic compilation of available database information. This model includes 5607 reactions and 2693 metabolites without any compartmentalization^18^.

In parallel to the bottom-up approach, the orthology-based method infers a genome-scale metabolic network of a target organism based on a previously reconstructed GSM of another reference organism^19,20^. The prerequisite is that the genomic sequence of a target organism shares a high degree of homology with its reference organism. Genes in the GSM of the reference organism could, therefore, be replaced by corresponding target organism orthologues. Several mammalian metabolic networks have been generated from different versions of human models as a reference^19–21^. Two existing mouse models, iMM1415^21^ and MMR^19^, were generated from human models of Recon1 and HMR2.0, respectively. Upon the release of a more recent version of reference GSM, corresponding target GSM models could be revisited and reconstructed once again.

Due to the metabolic differences between diverse mammalian tissues, a GSM model is applied to generate tissue-specific models via integration of multi-omics data. For this purpose, several algorithms such as GIMME^22^, INIT^23^, tINIT^24^ mCADRE^25^, FASTCORE^26^ have been developed. The constraint-based analysis of tissue-specific models has been widely applied to predict the metabolic phenotypes resulting from genetic and environmental perturbations and to identify potential biomarkers and drug targets^14,27–31^.

In this study, we took the advantages of the most recent human metabolic model, Recon3D^13^, to reconstruct a new and comprehensive metabolic model for mouse (*Mus musculus*). The orthology-based approach was adopted to map human genomic data of Recon3D to mouse. Two versions of the model were presented, both of which involving 1865 genes. A minimalist version, denoted as min-iMM1865, with a restriction on inclusion of non-gene-associated reactions, involves 8829 reactions, and the maximal version, denoted as iMM1865, involves 10612 reactions. Both models were validated against a set of 431 metabolic objective functions and used to construct a context-specific model of mouse embryo heart tissue based on expression array data and the metabolic Context-specificity Assessed by Deterministic Reaction Evaluation (mCADRE) algorithm^25^. Gene essentiality simulations were also used as another assessment of the predictive capabilities of reconstructed models. An extended mouse model denoted iMM3254 was also created from the extended version of Recon3D. Though it contains many dead-end and blocked components, it can be helpful in future reconstruction attempts.

## Results and Discussion

### Creating minimal and maximal mouse GSM models

**Figure 1:**
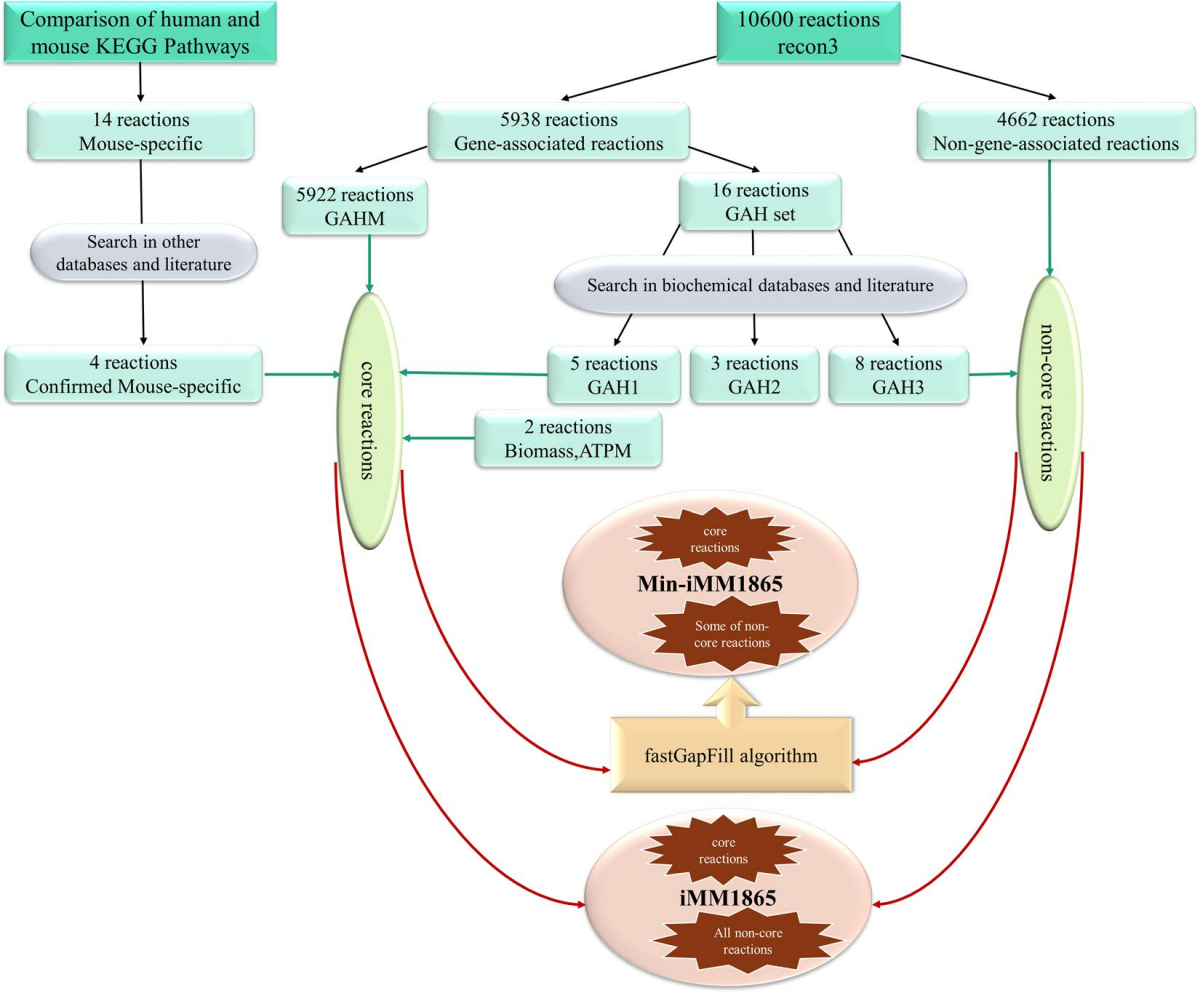
A workflow for the draft reconstruction of two versions of iMM1865 model: The process performs with determination of set of core reactions and set of non-core reactions based on Recon3D model and comparison of human and mouse KEGG pathways (see Result section for details). The set of core reactions consist of 1) 5922 gene-associated reactions in both human and mouse (GAHM set) 2) eight gene-associated reactions in human were decided to be in mouse after revising their corresponding GPR rules in Recon3D (GAH1 subset). 3) The mouse-specific reactions that were found using comparison of KEGG metabolism pathways of human and mouse, followed by searching in literature and databases. 4) Some of artificial reactions such as Biomass and ATP maintenance reactions. The set of non-core reactions consists of 1) all of non-gene-associated reactions in Recon3D 2) gene-associated reactions that were decided to be only in human (GAH3 subset). Finally, the min-iMM1865 model generated from the integration of set of core reactions and a minimal essential subset of non-core reactions that were selected using fastGapFill algorithm. The iMM1865 model generated from the integration set of core reactions and sum of all non-core reactions.

An overview of the adopted protocol for reconstruction of new mouse models is depicted in Fig. 1. It starts with the selection of Recon3D as the reference human model and maps the reference model genes to mouse orthologues. After manual curations and database and literature searches for mouse-specific components, two sets of core and non-core reactions were defined. These sets were subsequently combined into minimal and maximal versions of mouse models.

### Choosing Recon3D as a reference model

The well-known human GSM models are compared in Table 1. As can be seen, the most recent model, Recon3D, provides the most comprehensive set of reactions. However, there are two versions of Recon3D in Virtual Metabolic Human (VMH) database. The first version (denoted here as Recon3D_VMH) includes 13543 reactions, 8399 metabolites, and 3290 genes. The other version (referred here as Recon3D) corresponds to a subset of flux consistent reactions of Recon3D_VMH. It includes 10600 and 5835 reactions and metabolites, respectively. According to our goal which is reconstruction of a consistent mouse model, we have chosen Recon3D as a primary reference because it is more comprehensive and it has no dead-end metabolite or blocked reaction. To proceed with this model, all of its genes were searched in NCBI and some of them were found to be withdrawn. These genes and their GPR rules were removed from Recon3D. The other human model (Recon3D_VMH) was also used as a secondary reference to create an extended model that includes more genes and reactions but is not flux-consistent and has a lot of dead-end metabolites and blocked reactions.

**Table 1:** Comparison of well-known published human metabolic networks from three databases, VHM, BIGG, and HMR databases.

Models	Transcripts	Genes	Reactions	Metabolites	Dead-end Metabolites	Blocked-Reactions	Database	Publication Date	Reference
Recon1	1905	1496	3741	2766	342	1274	BIGG^1^	2007	^10^
HMR2.0	—	3765	8181	6006	499	608	HMR^2^	2014	^15^
Recon2.02	2191	1786	7440	5063	1178	1603	VMH^3^	2013	^12^
Recon3D_VMH	3697	3290	13543	8399	888	1582	VMH	2018	^13^
Recon3D_BIGG	2248	1884	10600	5835	0	0	BIGG	2018	^13^

^1^Biochemical, Genetic and Genomic knowledge base (http://bigg.ucsd.edu).

^2^The Human Metabolic Reaction (http://www.metabolicatlas.org/).

^3^Virtual Metabolic Human (https://vmh.life).

### Mapping Recon3D genes to mouse orthologues

Human genes in Recon3D were mapped to corresponding mouse orthologues by automatic application programming interface (API) programming complemented with a manual search of databases (see Supplementary Table-S1). The HomoloGene NCBI database and the orthologues reported in NCBI Gene records were searched by E-Utilities interface. This resulted in 1804 mouse orthologues out of 1884 Recon3D genes. The remaining 80 human genes were manually searched in KEGG orthology (KO) and Ensemble databases. This reduced the number of Recon3D human genes without any mouse orthologues to 43 (including 15 pseudogenes). For those human genes that were mapped to more than one mouse orthologues in HomoloGene database, further assessment was conducted by searching other databases (see Supplementary Table-S1).

### Compiling gene-product-reaction associations

Based on orthology relations between human and mouse genes, the set of Recon3D gene-product-reaction (GPR) rules were compiled to their corresponding logical expressions for the mouse model. Moreover, the existence or absence of mouse orthologues for human genes was interpreted as Boolean true/false values to divide Recon3D reactions into three different sets; 4662 non-gene-associated reactions, 5922 gene-associated reactions available in both human and mouse (GAHM set) and 16 gene-associated reactions in human (GAH set). An automatic gene orthology search provides no evidence for existence of a GAH set in the mouse model. However, this might be a result of a lack of information in Recon3D or searched databases. Accordingly, further databases and literature searches were performed before the final decision on each of these reactions.

Reassessment of Recon3D information and searching databases and literature divides the GAH set of reactions into three different subsets denoted as GAH1 to GAH3 in Fig. 1.

1. GAH1 subset consists of five reactions. The GPR rules of these reactions in Recon3D and their genes were revised and the reactions were kept in mouse model after repeating the orthologue mapping. For example, the reaction “2-Hydroxyphytanoyl Coenzyme A Lyase” (BIGG ID: HPCLx_1) in the fatty acid oxidation subsystem is associated with a single gene (Entrez gene ID: 26062) in Recon3D which was found to be a pseudogene and thus was not matched to any mouse orthologues. By correcting the association to a corresponding functional human gene (Entrez gene ID: 26061) and mapping it to the related mouse gene (Entrez gene ID: 56794) the reaction was decided to be present in both human and mouse (see Supplementary Note for description of all cases in GAH1 set).
2. It was decided that all three reactions in the GAH2 subset were to be removed from both human and mouse models. Because Recon3D model was still consistent after removal of these reactions (i.e. no reaction became blocked), they were not included in resulting mouse models. For example, the reaction “Mitochondrial GTP/GDP exchange carrier” (BIGG ID: R0801) is assigned to a single gene in Recon3D (*SLC25A6*, Entrez gene ID: 293) which corresponds to an ATP/ADP mitochondrial antiporter protein with no reported GTP/GDP transport activity. It should be noted that the same reaction corresponds to a multi-pass membrane protein in yeast (*GGC1*, Entrez gene ID: 851329). Unlike mammalian cells, in yeast cells, GTP is synthesized only outside the mitochondrial matrix in cytosol, so GTP/GDP transporter is necessary to regulate the level of these substances in yeast mitochondria^32,33^. On the other hand, the yeast *GGC1* gene has no reported human and mouse orthologue (see Supplementary Note for description of all cases in GAH2 set).
3. The GAH3 subset including eight reactions was decided to exist only in human and not in the mouse model since it lacked gene orthologues. Seven out of eight reactions were associated with three human alpha-(1, 3) fucosyltransferase genes (*FUT3*, *FUT5*, and *FUT6*) in Recon3D. The gene products involved in “Blood Group Synthesis” and “Glycosphingolipid” subsystems. The other remained human-specific reaction is retinyl ester hydrolase (BIGG ID: RETH) involved in “Vitamin A Metabolism” that catalyses the hydrolysis of retinyl ester to all-trans-retinol (vitamin A) plus a fatty acyl group^34,35^. The Recon3D gene associated  with this reaction is *PNPLA4* (Entrez gene ID: 8228) without any mouse orthologue in searched databases. However, since the consistency of Recon3D is influenced after removal of these human-specific reactions, they were kept as non-gene-associated reactions in subsequent steps of reconstruction (see Fig. 1 and also Supplementary Note for description of all cases in GAH3 set).

### Finding mouse-specific reactions

Without independent searching of mouse-specific metabolic functions, the reconstructed mouse model might be just a pruned version of a reference human model. To address the issue, comparison of human and mouse KEGG metabolic pathways was followed by searching other biochemical databases and literature. KEGG metabolic pathways of mouse contain fourteen reactions not presented in human pathways (see Supplementary Table-S2). Among these, only four reactions were considered as mouse-specific while there was no strong evidence in literature or other databases for others.

**Figure 2:**
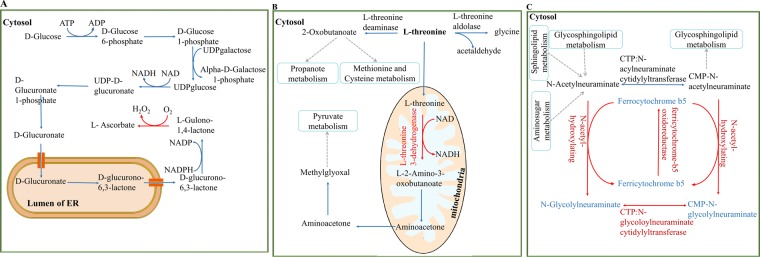
Mouse-specific reactions which were added into iMM1865. (**A**) Synthesis ascorbate (vitamin C) from glucose. The last enzyme of this pathway is not available in human. (**B**) There are three sub-pathways for threonine degradation. Human cells do not contain L-threonine 3-dehydrogenase enzyme. So breakdown of L-threonine into pyruvate does not occur. (**C**) A Sub-pathway of amino sugar metabolism which was added into iMM1865. Blue arrow: reactions exist in both Recon3D and iMM1865; Red arrow: reactions just exist in iMM1865; Dashed arrow: reactions are part of metabolic pathway; Black metabolites/enzymes: metabolites/enzymes exist in both Recon3D and iMM1865; Blue metabolites: metabolites just exist in iMM1865; Red enzymes: enzymes just exist in iMM1865. ER: endoplasmic reticulum.

The first reaction “L-gulono-1, 4-lactone: oxygen 3-oxidoreductase” (KEGG ID: R10053) catalysed by L-gulonolactone oxidase is the final step of the ascorbate biosynthetic pathway^36^. The *Glue* gene (Entrez gene ID: 268756) of mouse encodes L-gulonolactone oxidase. Human cells are not able to perform this reaction and produce vitamin C due to an absence of *Glue* orthologue^37,38^ (see Fig. 2 A). The second reaction “L-threonine: NAD + oxidoreductase” (KEGG ID: R01465) is oxidation of L-threonine by L-threonine dehydrogenase enzyme to L-2-Amino-3-oxobutanoate^39^. Mouse *Tdh* gene (Entrez gene ID: 58865) encodes this enzyme but Human *TDH* gene is an expressed pseudogene that encodes non-functional protein^40^ (see Fig. 2B).

Two last reactions, “N-acetylneuraminate, ferrocytochrome-b5:oxygen oxidoreductase” (KEGG ID: R01803) and “CMP-N-acetylneuraminate, ferrocytochrome-b5:oxygen oxidoreductase” (KEGG ID: R01115), are involved in aminosugar metabolism pathway (see Fig. 2C)^41^. Cytidine monophosphate-N-acetylneuraminic acid hydroxylase enzyme catalyses both of them which is encoded by the *Cmah* mouse gene (Entrez gene ID: 12763). The human *CMAH* gene differs from mouse *Cmah* by a deletion in a region corresponding to exon six of the mouse gene that causes an inactive hydrolase enzyme in human^42^. After addition of the last two reactions, it is required to also add four new metabolites including CMP-N-glycoloylneuraminate (BIGG ID: cmpglna), N-glycoloylneuraminate (BIGG ID: HC01115), ferricytochrome b5 (BIGG ID: ficytb5), and ferrocytochrome b5 (BIGG ID: focytb5) (see Fig. 2C).

### Creating maximal and minimal mouse models

Two different strategies were adopted that led to a maximal, larger mouse model and a minimal, smaller one. From a methodological point of view, one cannot prefer any of these with respect to the other but, as will be discussed later, these minimal and maximal models exhibit different levels of predictive capabilities in different contexts. Two sets of core and non-core reactions were defined. The core set includes all gene-associated reactions that should be available in both minimal and maximal models since there are well-known genomic evidence for these reactions formulated in their GPR rules. As can be seen in Fig. 1, the core set is a union of GAHM and GAH1 sets augmented with four mouse-specific and two artificial Biomass and ATPM reactions. The non-core set includes all non-gene-associated reactions of Recon3D and the GAH3 set of human reactions which are necessary to keep the consistency of Recon3D (see Supplementary Table-S3 for details about which reaction is in “core set” or “non-core set”).

In the minimal reconstruction approach, an initial draft was created from the core set of reactions. The core draft was then extended by adding a minimum number of non-core but necessary reactions to create a flux-consistent model (see Supplementary Table-S3). This goal was achieved by applying the fastGapFill algorithm which by design guarantees the minimalism of a resulting model. The initial minimal GSM mouse model which includes 8829 reactions and 5200 metabolites, will be referred to as min-iMM1865. On the other hand, the maximal approach combines both sets of core and non-core reactions to create a GSM model of 10612 reactions and 5839 metabolites, denoted as iMM1865. It should be noted that by excluding mouse-specific components both min-iMM1865 and iMM1865 inherit flux-consistency of the reference human model. The mouse-specific reactions in both models were also kept consistent by some additional sink reactions.

### Functionality tests of new mouse models

Since mice show high biochemical similarities with humans, the same metabolic functions can be used to test the functionality of mouse metabolic models. Similar validation strategies have been used in previous studies^20,21^.

Both versions of iMM1865 were evaluated against 431 metabolic objective functions which were developed to validate the functionality of Recon3D^13^. These functional tests were divided into two groups; 347 of them were metabolite conversion tests (MCTs) and 84 of them were reaction optimization tests (ROTs). In each MCT, the conversion of source metabolites to target metabolites, through one or multiple reactions, was tested under a RPMI1640 medium^43^ (see Methods section). For MCTs, iMM1865 and min-iMM1865 passed 322 and 312 tests, respectively, while iMM1415 and MMR passed 315 and 309 tests, respectively. In iMM1865, 19 tests failed because of missing source or target metabolites in the model. These numbers were 26, 1 and 29 for min-iMM1865, iMM1415 and MMR, respectively. Since most of failures in two iMM1865 versions were due to lack of source or target metabolites, we repeated the comparison with a list of tests whose participating metabolites are present in both versions of iMM1865. For this list (321 tests), iMM1865, min-iMM1865, iMM1415 and MMR passed 315, 312, 295 and 286 tests, respectively. For ROTs, iMM1865 and min-iMM1865 passed 79 and 62 tests compared to iMM1415 and MMR which passed 32 and 53 tests, respectively. Absence of some reactions leads to failure of 4, 16, 17 and 28 ROTs in iMM1865, min-iMM1865, iMM1415 and MMR, respectively. Among 68 tests, objective reactions were present in both versions of the presented models: iMM1865, min-iMM1865, iMM1415 and MMR passed 67, 62, 32 and 51 tests, respectively. The results clearly demonstrate considerable improvements of two versions of iMM1865 models compared to iMM1415 (see Supplementary Table-S4 for details).

### Comparison of iMM1865 and min-iMM1865 with previous mouse models

**Figure 3:**
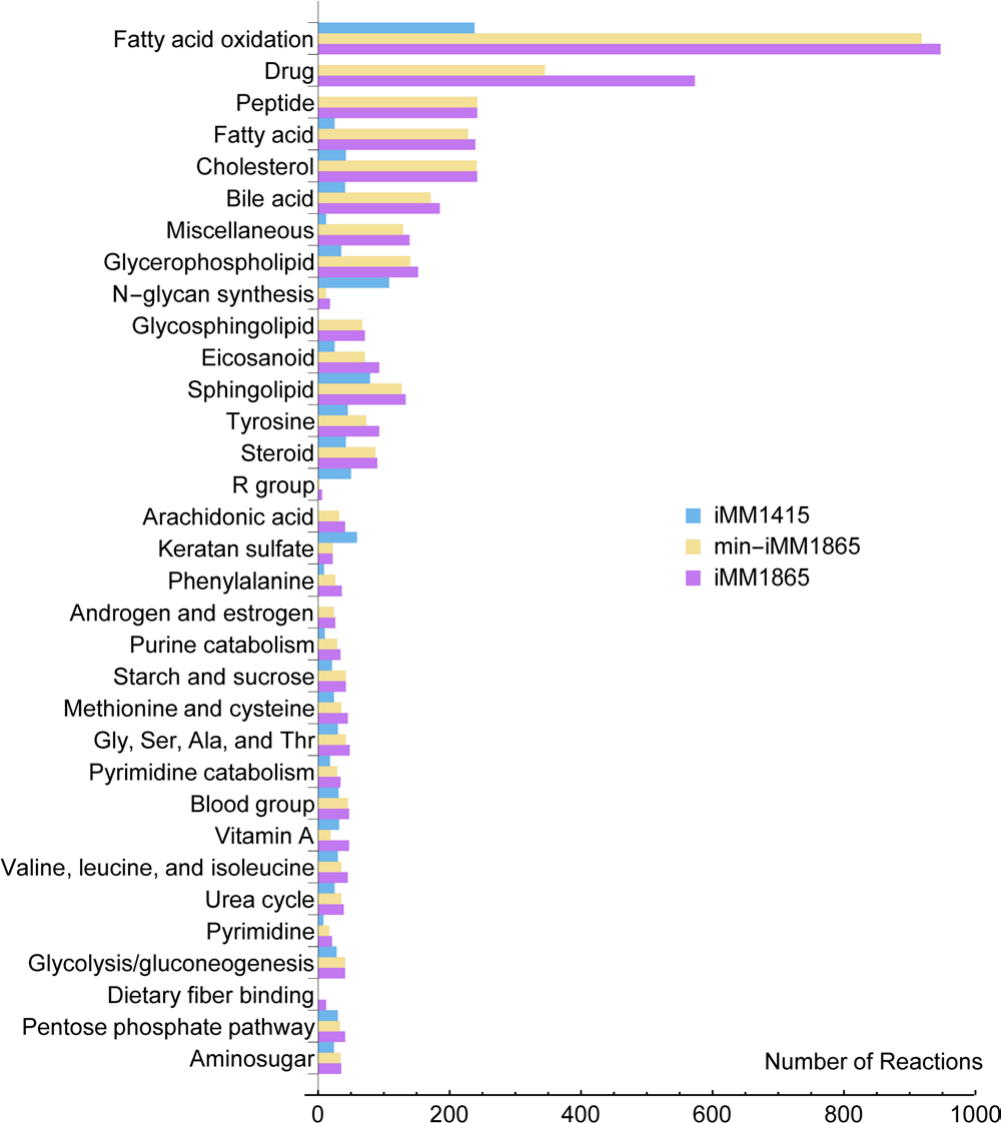
Comparison of subsystem distribution of reactions between iMM1865, min-iMM1865 and iMM1415.

Some features of iMM1865 and min-iMM1865 reconstructions are outlined in Table 2 and are compared with previously published mouse models. Regarding the number of reactions and metabolites, both versions of iMM1865 are more comprehensive than iMM1415 and MMR models. On the other hand, although MMR covers more gene-associated reactions, it has more blocked reactions; for example, there are many cases such as the “ATP: NAD + 2'-phosphotransferase” (KEGG id: R00104) and the “ATP: GMP phosphotransferase” (KEGG id: R00332) reactions, important to maintain the optimal ratio of NAD^+^/NADH and cellular GTP pool, respectively. These reactions carry flux in both iMM1865 versions but not in MMR and/or iMM1415 models. Furthermore, the MMR model, in contrast to the iMM1415 and iMM1865 models, has no information about enzyme complexes in corresponding GPR rules. For both versions of iMM1865 and iMM1415 models, distribution of reactions within subsystems is shown in Fig. 3. Only those subsystems with more differences between iMM1865 and iMM1415 are considered in Fig. 3 (see Supplementary Table-S5 for the full list). The reactions involved in “Drug Metabolism” and “Peptide Metabolism” pathways were included in iMM1865 models, while they are absent in iMM1415. The lipid metabolism pathways such as “Fatty Acid Oxidation”, “Cholesterol Metabolism” and “Fatty Acid Synthesis” in iMM1865 are more comprehensive than iMM1415. For different pathways of lipid metabolism in two versions of iMM1865 model, more than 1800 reactions are involved, compared to nearly 500 reactions in iMM1415. Such a difference implies the advantage of studying lipid metabolism in iMM1865. In contrast, iMM1415 model encompasses a larger number of reactions for “N-glycan Synthesis” subsystem.

**Table 2:** Different versions of iMM1865 model and their comparison with the previously published models.

Models	Published Models				Current Models	
	Quek and Nielsen	Selvarasu	iMM1415	MMR	Min-iMM1865	iMM1865
Genes	1399	724	1375	3579	1865	1865
Reactions	2037	1494	3726	8140	8829	10612
Gene-Associated Reactions	1889	1203	2210	6005	5931	5931
Metabolites	2110	1162	2775	5993	5200	5839
Blocked Reactions	1176	—	1276	329	0	0
Metabolite Objective Functions	—	—	347	362	374	401

### Gene knock-out simulations

**Figure 4:**
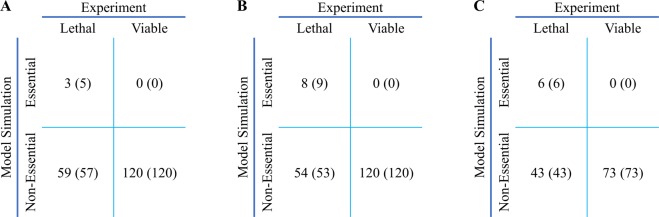
The confusion matrix for comparison between in silico predictions of gene essentiality vs. experimental results in two versions of iMM1865 and iMM1415 (based on FBA and linearMOMA approaches). (**A**) The confusion matrix of iMM1865 model based on FBA and lMOMA (placed in parentheses) methods. (**B**) The confusion matrix of min-iMM1865 model based on FBA and lMOMA (placed in parentheses) methods. (**B**) The confusion matrix of iMM1415 model based on FBA and lMOMA (placed in parentheses) methods. A gene is essential if its deletion reduces the biomass objective value less than 30% of the optimal value in wild type. The sensitivity score for iMM1865, min-iMM1865 and iMM1415 are 5%, 13% and 12% based on FBA method. The sensitivity score for iMM1865, min-iMM1865 and iMM1415 are 8%, 14% and 12% based on lMOMA method. The specificity score for iMM1865, min-iMM1865 and iMM1415 are 100% based on both FBA and lMOMA methods.

A common approach to evaluate metabolic models is using them to simulate effects of single gene knock-outs on growth rates and compare predicted values with available experimental results. Flux Balance Analysis (FBA)^44^ and linear version of Minimization Of Metabolic Adjustment (lMOMA)^45^ methods were used to perform gene knock-out simulations for 62 lethal (see Supplementary Table-S6) and 120 viable (see Supplementary Table-S7) metabolic genes in iMM1865 and min-iMM1865, iMM1415 models under HAM media^24^. An essential gene is a gene whose deletion reduces biomass production to less than a significant fraction (30%) of its optimal value in a wild type model. Linear MOMA approach in min-iMM1865 has better compatibility with experimental results compared with the other simulations on genome-scale models (see Fig. 4). It should be noted that iMM1415 does not include all of the experimentally tested genes. It just contains 49 (out of 62) lethal and 73 (out of 120) viable genes. Both iMM1865 and min-iMM1865 as GSM models include all reactions that take place in mice in different external and internal conditions and different tissues. In these GSM models, removal of a reaction is less likely to significantly decrease the biomass production since alternative pathways or isozymes exist and they compensate for the effect of considered knock-out. All isozymes of an enzyme may not be active in different tissues; for example, lactate dehydrogenase (LDH), a major enzyme in anaerobic glucose metabolism, has five isozymes in which LDH-5 (M4) is the major isozyme of skeletal muscle and liver, whereas LDH-1 (H4) is the main isozyme for heart muscle^46^. So, the deletion of genes that encode isozymes has tissue-specific consequences. Also, the alternative pathways might not be active under certain conditions or in a specific context. To improve the prediction ability of the models, we reconstructed tissue-specific models from the GSM models. In recent years, several algorithms have been proposed to reconstruct tissue-specific metabolic models based on a GSM model. Since the lethal and viable metabolic gene lists are results of an experimental investigation of gene knock-outs in embryo mouse, an embryonic tissue for which more genes were found to be expressed were selected. We generated heart embryonic model from iMM1865, min-iMM1865 and iMM1415 using the mCADRE algorithm^25^ and gene expression data. We downloaded 139 microarray samples of embryo hearts from the GEO database. After removing outlier samples, 119 of the remaining arrays (see Supplementary Table-S8) were binarized using the Affymetrix MAS5 detection call^47^. The mCADRE algorithm uses the binarized expression data in conjunction with network topology features to prune the global GSM model and reconstruct the embryo heart model. Based on different selected thresholds for expression-based score in mCADRE, we reconstructed three embryo heart models from each GSM model (see Table 3). As mentioned before, we performed gene knock-out simulations for the embryo heart models and summarized results in Table 4. It should be noted that the results of different types of embryo heart models are in better agreement with experimental results compared to the GSM models. This improvement in prediction in embryo heart networks as compared to the GSM models might be due to the elimination of alternative reactions or pathways. iMM1865 (lMOMA) has an 8% sensitivity and a 100% specificity while iHeart1653 (lMOMA) has a 23% sensitivity and a 98% specificity. These numbers highlight the improvement in sensitivity (true positives for lethal genes) with the iHeart model in agreement with our hypothesis about the role of the elimination of alternative reactions or pathways. Again, a better prediction was achieved, using linear MOMA approach than FBA method for embryo heart models, derived from min-iMM1865 especially when one considers the fact that iMM1415 supports a smaller number of experimentally assessed genes.

**Table 3:** Different versions of embryo heart generated from iMM1865, min-iMM1865 and iMM1415.

Model	Reactions	Metabolites	Genes
iHeart1709^1^	7080	4589	1709
iHeart1696^1^	6944	4552	1696
iHeart1653^1^	5784	3949	1653
Min-iHeart1716^2^	7090	4608	1716
Min-iHeart1703^2^	6941	4567	1703
Min-iHeart1651^2^	5786	3972	1651
iHeart1041^3^	1868	1453	1041
iHeart1034^3^	1843	1444	1034
iHeart983^3^	1705	1373	983

^1^iHeart1709, iHeart1696, and iHeart1653 reconstructed based on iMM1865 through mCADRE algorithm with a threshold of 0.5, 0.7, and 0.9 for expression-based score, respectively.

^2^min-iHeart1716, min-iHeart1703, and min-iHeart1651 reconstructed based on min-iMM1865 through mCADRE algorithm with a threshold of 0.5, 0.7, and 0.9 for expression-based score, respectively.

^3^iHeart1041, iHeart1034, and iHeart983 reconstructed based on iMM1415 through mCADRE algorithm with a threshold of 0.5, 0.7, and 0.9 for expression-based score, respectively.

**Table 4:** Classification of results for gene knock-out simulation in different versions of embryo heart generated from iMM1865, min-iMM1865 and iMM1415 based on FBA and linear MOMA approaches.

		TP	FP	FN	TN	Sensitivity	Specificity
FBA method	iHeart1709	9	1	53	119	15%	99%
	iHeart1696	9	1	53	119	15%	99%
	iHeart1653	13	2	49	118	21%	98%
	min-iHeart1716	9	1	53	119	15%	99%
	min-iHeart1703	9	1	53	119	15%	99%
	min-iHeart1651	13	1	49	119	21%	99%
	iHeart1041	9	5	40	68	18%	93%
	iHeart1034	9	5	40	68	18%	93%
	iHeart983	9	5	40	68	18%	93%
lMOMA method	iHeart1709	10	1	52	119	16%	99%
	iHeart1696	10	1	52	119	16%	99%
	iHeart1653	14	2	48	118	23%	98%
	min-iHeart1716	10	1	52	119	16%	99%
	min-iHeart1703	10	1	52	119	16%	99%
	min-iHeart1651	14	1	48	119	23%	99%
	iHeart1041	9	5	40	68	18%	93%
	iHeart1034	9	5	40	68	18%	93%
	iHeart983	9	5	40	68	18%	93%

**TP**: True Positive, **FP**: False Positive, **FN**: False Negative, **TN**: True Negative.

**Sensitivity**= TP/(TP+FN), **Specificity**=TN/(TN+FP).

### The extended mouse model

Up to this point, both min-iMM1865 and iMM1865 are flux-consistent functional models suitable for many applications and simulations via constraint-based modelling. However, they do not reflect all of our knowledge about mouse metabolism. There are many more reactions in the extended version of Recon3D (Recon3D_VMH) and it might be interesting to know which of them can be mapped to the mouse. In this regard, we repeated the above mentioned orthology based procedure by starting from Recon3D_VMH to create an extended mouse model denoted as iMM3254. It contains 13543 reactions (7666 gene-associated), 8395 metabolites and 3254 genes. It is obvious that this model is a much more comprehensive but with 884 dead-end metabolites and 1569 blocked reactions. The functional tests also performed on this model. From 347 MCTs only five tests failed due to non-optimality. From 84 ROTs, three of them failed because corresponding reactions are not available in the model and another test failed due to non-optimality. In the light of ongoing increase in our knowledge about metabolism of human and mouse, iMM3254 can be a suitable starting point for future developments in creating better mouse models.

## Conclusion

In this study, we have prepared two versions of orthology-based reconstructed *Mus musculus* metabolic model. In iMM1865 models, we replaced genes of human metabolic Recon3D with their corresponding *Mus musculus* orthologues. The difference between the two versions is the availability of non-gene-associated reactions in the final model. In the minimal version (min-iMM1865), the final model only consists of a small subset of these reactions which are essential for model consistency. In another approach, the maximal version of the model (iMM1865) was obtained by the inclusion of all non-gene-associated reactions. To avoid the new mouse models being just a pruned version of the reference model, mouse-specific reactions were also added to both reconstructed versions. The mouse-specific reactions were extracted through comparison of KEGG metabolic networks of human and mouse and also were searched by other biochemical databases and literature. These models have been validated using 431 metabolic objective functions which have been used to verify Recon3D^13^. The iMM1865 and min-iMM1865 models satisfied 401 and 374 of these tests, respectively, which is an improvement over the previously published mouse models, iMM1415 and MMR, that only passed 347 and 362 tests, respectively.

Moreover, two versions of iMM1865 have more comprehensive annotations of metabolite and reactions compared to iMM1415. Unlike iMM1415, iMM1865 covers reactions and metabolites that are involved in “Drug Metabolism” and “Peptide Metabolism” pathways. In addition, the reconstruction of lipid metabolism pathways is more complete in iMM1865 compared with iMM1415. Therefore, it is a more appropriate model to study mouse lipid metabolism. Two versions of iMM1865 models unlike iMM1415 do not have any dead-end metabolites and blocked reactions.

In comparison with the other published mouse model MMR, iMM1865 models are flux consistent, without including any blocked reactions and dead-end metabolites. Also, the GPR rules of MMR model, unlike iMM1865 models, did not include any information related to enzyme complexes which is very important for mapping omics data to GSM models.

We have used FBA and a linear version of MOMA to predict gene essentiality in iMM1865, min-iMM1865 and iMM1415. The essentialities of mouse metabolic genes were predicted based on a significant reduction of growth rate. For this purpose, we have simulated gene knock-out analysis for the experimentally reported data including a list of lethal and viable genes. The best prediction was for the min-iMM1865 model with the lMOMA method. Since the experimental data were phenotypes resulting from knock-outs of genes in mouse embryo, we also repeated the simulations for embryonic tissue-specific models. We reconstructed embryo heart models from iMM1865, min-iMM1865 and iMM1415 using mCADRE algorithm. Our findings suggest that tissue-specific models have better predictions than GSM models. The perturbation caused by deletion of a gene in a GSM model could be damped due to the existence of alternative pathways or isozymes while these pathways or isozymes might be inactive or absent in the tissue-specific model resulting in better agreement with gene essentiality experiments.

To date, the two versions of iMM1865 are the most comprehensive consistent models of mouse metabolism. Different types of context-specific models can be reconstructed using integration of multi-omics data into these GSM models. These models allow prediction of behaviour of the organism in response to environmental and genetic perturbations. Since mouse is a model organism for human biology, these models can be used to identify candidate metabolic biomarkers and drug effects for diseases such as cancer and complex metabolic disorders.

## Methods

### Reconstruction procedure

Two versions of iMM1865 were reconstructed using COBRA toolbox v3.0^48^. They were reconstructed from Recon3D as the largest reported human metabolic model without blocked reactions. Recon3D consists of gene-associated and non-gene-associated reactions. For every gene-associated reaction, relationships between genes and reaction are represented by a Gene-Protein-Reaction (GPR) logical rule. To reconstruct mouse genome-scale metabolic network from Recon3D, human genes in the GPR rules were replaced with mouse orthologues. HomoloGene and orthologues mouse records in NCBI’s Gene database were used for identification of mouse orthologous. Entrez Programming Utilities (E-utilities) provide the programmatic access to these databases. If a search found no match, KEGG orthology and Ensemble databases were searched for missing orthologous genes. Gene-associated reactions of Recon3D that are affected by missing orthologues genes were further investigated in other biochemical databases and literature and categorized into three groups. Mouse-specific reactions were determined using a comparison of human and mouse metabolic KEGG pathways and literature reviews. The KEGG API^49,50^ was used to obtain human and mouse biochemical pathways.

We have defined all mouse gene-associated reactions as a set of core reactions and all non-mouse gene-associated reactions as a set of non-core reactions. Two strategies were applied for the addition of non-core reactions to the set of core reactions in order to reconstruct final genome-scale models. In the first strategy, only non-gene-associated reactions that are essential for carrying flux over core reactions were added to the core set. We used fastGapFill algorithm^51^ to select a minimum set of these reactions. The fastGapFill function is a COBRA toolbox extension to find candidate missing reactions from reference databases such as KEGG for a desired metabolic model. Herein, we have applied this function to identify missing reactions in the set of core reactions from a set of non-core reactions. In the second strategy, all non-gene-associated reactions were added to the set of core reactions and also included in the final model. All models generated in this study can be found in supplemental data as SBML files.

### Validation of reconstructed models

During the draft reconstruction process, fastFVA^52^ was performed to determine reactions with zero flux. fastFVA is an implementation to perform more efficiently the flux variability analysis (FVA)^53^ which calculates the minimum and maximum ranges of fluxes through each reaction in the metabolic model. To evaluate the functionality of the reconstructed models, we used 431 metabolic functional tests previously introduced for validating Recon3D^13^. These tests can be categorized into two groups; metabolite conversion tests (MCTs) and reaction optimization tests (ROTs). In the first group, conversion of some metabolites (source metabolites) to some other metabolites (target metabolites) was tested under RPMI1640 medium^43^. Source metabolites can be converted to target metabolites through one or multiple reaction steps; for example, for the conversion test of glucose to pyruvate, a series of reactions involved in glycolysis pathway must be included in a model to break down glucose to pyruvate. To test this function, first, reactions were added (if not available) to represent a source of glucose and consumption of pyruvate, while all uptake reactions except those for RPMI1640 medium were set to be closed. Then, the ability of pyruvate production through glucose was checked by maximizing the flux through pyruvate demand reaction. In the second group, ROTs, a reaction was checked for its availability and ability to carry non-zero flux under specific conditions; for example, we analysed the model for maximum production of ATP from glucose under aerobic conditions. First, we closed all exchange reactions of organic compounds except glucose and then maximized ATP maintenance reaction (ATPM) as an objective reaction. It should be noted that under aerobic conditions, Oxygen uptake is allowed while its uptake will be prevented under anaerobic conditions. There are three types of failure for MCTs and ROTs. The MCT will fail when *i*) the source or target metabolites do not exist in the model, *ii*) the optimal solution of the test objective does not exist, or *iii*) the value of the test objective is almost zero. The ROT will fail if *i*) the test reaction does not exist in the model, *ii*) optimal solution does not exist for the test reaction as an objective, *iii*) the value of the test objective is almost zero.

### Transcriptomic data

To reconstruct a tissue-specific metabolic model using mCADRE, we downloaded raw gene expression microarray profiling data from gene expression omnibus (GEO) database^54^. The data consists of 139 samples derived from healthy hearts at different embryonic stages using GPL1261 platform. Prior to data binarization, we performed pre-processing to identify and remove outlier samples. The Pearson correlation coefficient (PCC) was calculated between every pair of samples. The following standard score formula was used to determine outlier samples:^55^

$$Zscore=\frac{{x}_{i}-\bar{x}}{s}$$

where ${x}_{i}$ is the mean of the PCC for each array sample, $\bar{x}$ is the mean of all calculated PCCs, and *s* is standard deviation. Samples with two and more standard deviation values above or below the total mean were detected as outliers and removed. The process of outlier detection was repeated until all samples with divergent gene expression pattern were ruled out. Finally, 119 microarray samples were used to generate embryo heart model (see Supplementary Table-S8).

The Affymetrix GPL1261 platform has 45101 probe sets that map to 21603 unique genes. A binary matrix represents the state of expression of each gene in a particular sample. We used “mas5call” algorithm^47^ in affy package of BioConductor to flag each gene as “Present”, “Marginal”, and “Absent” (P/M/A). For gene binarization, “Present”, “Marginal”, and “Absent” calls replaced with 1, 0, and 0, respectively. For genes with multiple probe sets, the maximum expression value was used.

### Utilizing modified mCADRE algorithm

The mCADRE algorithm categorizes GSM reactions into two sets, core and non-core set reactions, using an expression-based score. The reaction expression-based score was determined by the frequency of expressed state of genes that were associated with a desired reaction across expression profiles of the selected tissue. The binarized expression data was used to calculate the frequency of expressed states of each gene. A reaction with a high expression-based score has strong evidence to be active in the desired tissue. The core set reactions have an expressed-based score at or above the fixed selected threshold. The non-core set reactions were ranked based on expressed-based and connectivity-based scores. The connectivity-based score for each reaction is a parameter of network topology that estimates connectivity of all its adjacent reactions. In this study, due to the unavailability of reaction biological evidence data, the confidence score was ignored to rank non-core reactions. The mCADRE algorithm sequentially removes ordered non-core reactions from the GSM model. At each iteration of the algorithm, the functionality of the model for key metabolites produced from glucose as well as model consistency were checked. A reaction was eliminated if it did not affect the functionality and consistency of the model. In this study, a new function was added to mCADRE in order to test biomass production under HAM media^24^ during the model building process. If removal of a reaction reduced the objective value of biomass reaction less than 80% of the value of the GSM model, the reaction would remain in the final tissue-specific model. Similar to *checkModelFunction* and *checkModelConsistency* built-in functions, *checkBiomassProduction* function was also checked in every iteration.

### Gene knock-out experiment data

The embryo’s phenotypes resulting from 1751 unique gene knock-outs were reported in 2016^56^. This report identified 410 homozygote lethal genes and 1143 homozygote viable genes. The number of 62 metabolic lethal genes (see Supplementary Table-S6) and 120 metabolic viable genes (see Supplementary Table-S7) which were also available in the reconstructed models were selected to validate prediction capabilities of the models.

### Computational methods for simulation of gene knock-out

The FBA^44^ problem was formulated to find a flux distribution that maximizes biomass production in gene knock-out model under HAM media^27^.

1 $$\max \,{\upsilon }_{biomass}$$

2 $$s.\upsilon =0$$

3 $$-1000\le {\upsilon }_{i}\le 1000,$$

4 $$\begin{array}{c}i\in Exchange\,reactions\,indexes\,for\,HAM\,media\,metabolites\\ {\upsilon }_{r}=0,\\ r\in reactions\,associated\,with\,the\,deleted\,gene\end{array}$$

where ${\upsilon }_{biomass}$ is a flux through biomass reaction. Equation 2 represents mass balance constraints, where $s$ is stoichiometry matrix containing a stoichiometric coefficient of each metabolite in every reaction and $\upsilon$ is a vector of fluxes through all reactions in a metabolic model. Based on Eq. 3, the flux range for exchange reactions corresponding to HAM media composition were between −1000 and +1000, while flux ranges of other exchange reactions were set to zero in the model. In Eq. 4, upper and lower flux bounds of the reactions affected by deleted gene were constrained to zero^27^.

The method of the Minimization of Metabolic Adjustment (MOMA)^45^ is another approach to predict phenotypic consequences of genetic perturbation in a metabolic network. MOMA can be used to identify the closest sub-optimal flux distribution following a perturbation to an optimal wild-type solution. A linear version of MOMA is formulated as follows:

5 $$min{\sum }^{}|{v}_{w}-{v}_{d}|$$

6 $${S}_{w}{v}_{w}=0$$

7 $$l{b}_{w}\le {v}_{w}\le u{b}_{w}$$

8 $${c}_{w}^{T}{v}_{w}={f}_{w}$$

9 $${S}_{d}{v}_{d}=0$$

10 $$l{b}_{d}\le {v}_{d}\le u{b}_{d}$$

where MOMA optimization minimizes the absolute value of distance between wild type and perturbed flux distribution. Equations 6 and 7 represent mass balance constraints for wild type and perturbed models, where ${s}_{w}$ and ${s}_{d}$ are stoichiometry matrix of wild type and perturbed models, respectively, and ${v}_{w}$ and ${v}_{d}\,$are vectors of flux distribution for wild type and perturbed models, respectively. Based on Eq. 9, ${f}_{w}$ is an optimal wild type objective value which is selected as a starting point for MOMA optimization instead of a random starting point. All organic exchange reactions of model, except exchange reactions corresponding to HAM media composition, are closed. *singleGeneDeletion* function in COBRA toolbox was applied for gene knock-out simulation. Three methods consist of FBA, quadratic version of MOMA approach, and a linear version of MOMA (lMOMA) were implemented in *singleGeneDeletion* function to perform single gene deletion analysis.

### Validation of predicted gene essentiality

Results of gene knock-out prediction fall into four groups: True Positives (TP), True Negatives (TN), False Positives (FP), and False Negatives (FN). TP means a lethal gene was correctly predicted as an essential gene. TN indicates a viable gene was correctly predicted as a non-essential gene by the model. FP means the model incorrectly predicted a viable gene as an essential gene. Finally, FN indicates the model incorrectly predicted a lethal gene as a non-essential gene. Using sensitivity and specificity, the performance of gene knock-out simulation tests was evaluated. The sensitivity of a test calculates the proportion of actual essential genes that are correctly predicted. The specificity of a test reflects the proportion of actual non-essential genes that are correctly predicted. The mathematical representation of sensitivity and specificity are as follows:

11 $$Sensitivity=TP/(TP+FN)$$

12 $$Specificity=TN/(TN+FP)$$

## Supplementary information

Supplementary Note.

Supplementary Data 1.

Supplementary Data 2.

Supplementary Data 3.

Supplementary Data 4.

Supplementary Data 5.

Supplementary Data 6.

Supplementary Data 7.

Supplementary Data 8.

Supplementary Data 9.

Supplementary Table-S1.

Supplementary Table-S2.

Supplementary Table-S3.

Supplementary Table-S4.

Supplementary Table-S5.

Supplementary Table-S6.

Supplementary Table-S7.

Supplementary Table-S8.
