# Supplementary material for: iMM1865: A New Reconstruction of Mouse Genome-Scale Metabolic Model
Source: Sci Rep. 2020 Apr 10;10:6177. doi: 10.1038/s41598-020-63235-w (PMC7148337; doi:10.1038/s41598-020-63235-w)
Supplement: Supplementary file 1 — Supplementary Note. [file 41598_2020_63235_MOESM1_ESM.docx]

**iMM1865: A New Reconstruction of Mouse Genome-Scale Metabolic Model**

Saeideh Khodaee^1^, Yazdan Asgari^2^, Mehdi Totonchi^3,4^, Mohammad Hossein Karimi-Jafari^1*^

^1^ Department of Bioinformatics, Institute of Biochemistry and Biophysics, University of Tehran, Tehran, Iran

^2^ Department of Medical Biotechnology, School of Advanced Technologies in Medicine, Tehran University of Medical Sciences, Tehran, Iran

^3^ Department of Stem Cells and Developmental Biology, Cell Science Research Center, Royan Institute for Stem Cell Biology and Technology, ACECR, Tehran, Iran

^4^ Department of Genetics, Reproductive Biomedicine Research Center, Royan Institute for Reproductive Biomedicine, ACECR, Tehran, Iran

Correspondence and requests for materials should be addressed to M. H. K-J. (Email: [mhkarimijafari@ut.ac.ir](mailto:mhkarimijafari@ut.ac.ir) )

**Set of the gene associated reactions in human (GAH)**

1. Reactions of GAH1 set

The reaction “2-Hydroxyphytanoyl Coenzyme A Lyase” (BIGG ID: HPCLx_1) in the fatty acid oxidation subsystem is associated to a single gene (Entrez gene ID of 26062) in Recon3D which found to be a pseudogene thus not matched to any mouse orthologue. By correcting the association to the corresponding functional human gene (Entrez gene ID of 26061) and mapping to related mouse gene (Entrez gene ID of 56794) the reaction was decided to be in both human and mouse.

During “P4503A43r” reaction, Testosterone convert to 19-Hydroxytestosterone using NADPH-hemoprotein as electron donor and oxygen. Based on the BIGG models of human and mouse, this reaction is catalysed by unspecific monooxygenase (EC: 1.14.14.1) but in other major enzyme reaction databases such as KEGG (R02501), RHEA (38182) and BRENDA (EC1.14.14.14) databases catalysed by Aromatase (EC: 1.14.14.14). We consider that Aromatase catalyse this reaction and update corresponding GPR to Entrez gene ID of “13075” in new models.

The “PNTEH” reaction catalysed by pantetheine hydrolase that encoded by three genes in human (*VNN1, VNN2, VNN3*) and two genes in mouse (*Vnn1*, *Vnn3*). GPR associated with this reaction in IMM1415 is “26464 AND 22361 AND 55350” that “55350” is human GeneID. In iMM1865 we update it to “26464 AND 22361”.

The “DHEASABCCte” and the “ESTROSABCCte” are two transporter reactions in Recon3D that transport DHEAS and Estrone 3-sulfate from extracellular to cytosol, respectively. The gene associated to these transporter reactions is *ABCC11* (Entrez gene ID of 85320) that encodes a member of the superfamily of ATP-binding cassette (ABC) transporters involved in uptake of synthetic lipophilic anions, steroid sulfates such as dehydroepiandrosterone 3-sulfate (DHEAS) and estrone 3-sulfate and glucuronides^1^. The *ABCC11* gene has no orthologue in mouse. Using search in literature, we found that steroid sulfates also are substrates for ABCC4 protein^2^. The mouse gene orthologue of *Abcc4* (geneID: 239273) had been assign to these transporter reactions.

1. Reactions of GAH2 set

The reaction “Mitochondrial GTP/GDP exchange carrier” (BIGG ID: R0801) is assigned to a single gene in Recon3D (*SLC25A6*, Entrez gene ID of 293) which corresponds to an ATP/ADP mitochondrial antiporter protein with no reported GTP/GDP transport activity. It should be noted that the same reaction corresponds to a multi-pass membrane protein in yeast (*GGC1*, Entrez gene ID of 851329). Unlike mammalian cells, in yeast cell, GTP is synthesized only outside the mitochondrial matrix in cytosol, so GTP/GDP transporter is necessary to regulate the level of these substances in yeast mitochondria^3,4^. Moreover, the yeast *GGC1* gene has no reported human and mouse orthologue.

The “Phosphoadenylyl-sulfate reductase” reaction (PAPSR) is assigned to *SULT1A3* (Entrez gene ID of 1688) that encodes an aryl sulfotransferase (EC: 2.8.2.1). Unlike Recon3D model, based on other databases (KEGG (R02021), RHEA (11727) and MetaCyc (1.8.4.8-RXN) databases) the PAPSR reaction is catalysed by phosphoadenylyl-sulfate reductase enzyme (EC: 1.8.4.8). This enzyme was found in neither human nor mouse, so this reaction was not included in iMM1865 models.

The “2-Phenylacetamide Amidohydrolase” (HMR_7756) is assigned to *FAAH2* gene (Entrez gene ID of 158584) in Recon3D. This reaction correspond to R02540 record in KEGG database that catalysed by amidase (EC: 3.5.1.4). Since this enzyme was found in neither human nor mouse, we have not included it in iMM1865 models.

1. Reactions of GAH3 set

The “Patatin like phospholipase domain containing 4” (PNPLA4) catalyses the hydrolysis of retinyl ester to All-trans-Retinol (vitamin A) plus a fatty acyl group^5,6^. There is no homolog of this gene in the mouse genome, so we consider this reaction as a human-specific reaction.

Seven of these reactions FUT32g, FUT33g, FUT34g, FUT35g, HMR_0870, HMR_ 0873 and HMR_ 8271 were associated in Recon3D to three human alpha-(1, 3) fucosyltransferase genes (*FUT3*; Entrez gene ID of 2525, *FUT5*; Entrez gene ID of 2527, and *FUT6*; Entrez gene ID of 2528). Since the corresponding mouse orthologues of these genes are not reported, we consider these reactions as a human-specific reactions.

**References:**

1. Chen, Z.-S. Transport of Bile Acids, Sulfated Steroids, Estradiol 17- -D-Glucuronide, and Leukotriene C4 by Human Multidrug Resistance Protein 8 (ABCC11). *Mol. Pharmacol.* **67**, 545–557 (2004).

2. Zelcer, N. *et al.* Steroid and bile acid conjugates are substrates of human multidrug-resistance protein (MRP) 4 (ATP-binding cassette C4). *Biochem.J.* **371**, 361–367 (2003).

3. Gordon, D. M., Lyver, E. R., Lesuisse, E., Dancis, A. & Pain, D. GTP in the mitochondrial matrix plays a crucial role in organellar iron homoeostasis. *Biochem. J.* **400**, 163–168 (2006).

4. Vozza, A., Blanco, E., Palmieri, L. & Palmieri, F. Identification of the mitochondrial GTP/GDP transporter in Saccharomyces cerevisiae. *J. Biol. Chem.* **279**, 20850–20857 (2004).

5. Gao, J. G. & Simon, M. Molecular Screening for GS2 Lipase Regulators: Inhibition of Keratinocyte Retinylester Hydrolysis by TIP47. *J. Invest. Dermatol.* **126**, 2087–2095 (2006).

6. Gao, J. G. & Simon, M. A comparative study of human GS2, its paralogues, and its rat orthologue. *Biochem. Biophys. Res. Commun.* **360**, 501–506 (2007).
